# Plasma-Methylated SEPT9 for the Noninvasive Diagnosis of Gastric Cancer

**DOI:** 10.3390/jcm11216399

**Published:** 2022-10-29

**Authors:** Luyao Zhao, Muran Li, Shiwu Zhang, Yandi Liu

**Affiliations:** 1 Department of Gastroenterology, Tianjin Union Medical Center, Tianjin 300121, China; 2 Department of Pathology, Tianjin Union Medical Center, Tianjin 300121, China

**Keywords:** diagnosis, DNA methylation, gastric cancer, SEPT9

## Abstract

Background. Gastric cancer (GC) is one of the most prevalent cancers globally. This study was designed to evaluate the potential performance of plasma SEPT9 methylation (mSEPT9) as a noninvasive biomarker for the diagnosis of GC. Methods. A total of 182 participants, i.e., 60 patients with GC, 39 with chronic superficial gastritis (CSG), 27 with chronic atrophic gastritis (CAG), 30 with gastric ulcer (GU), and 26 with gastric polys (GP), were recruited. The mSEPT9 level was measured using real-time polymerase chain reaction. Results. As a diagnostic target, mSEPT9 (1/3 algorithm) had a sensitivity of 48.33 (95% confidence interval (CI): 35.40–61.48%) and a specificity of 86.89% (95% CI: 79.28–92.09%), and mSEPT9 (2/3 algorithm) had a sensitivity of 33.33 (95% CI: 22.02–46.79%) and a specificity of 98.36% (95% CI: 93.61–99.72%). The area under the receiver operating characteristic curve (ROC) curve of mSEPT9 was 0.698 (95% CI: 0.609–0.787) for the differentiation of GC from benign gastric diseases. The effectiveness of mSEPT9 (1/3 algorithm) was superior to that of CEA, CA19-9, and CA72-4. mSEPT9 was positively correlated with T, N, M, and the clinical stage of GC. Conclusions. Plasma mSEPT9 might serve as a useful and noninvasive biomarker for the diagnosis of GC.

## 1. Introduction

Gastric cancer (GC) is one of the most common malignant tumors worldwide. According to an epidemiologic study, GC is currently the fifth most common malignant disease, but it is the fourth leading cause of cancer-related death [1]. Intestinal-type GC is characterized by chronic inflammation, chronic atrophic gastritis (CAG), intestinal metaplasia, dysplasia, and GC [2]. The World Health Organization regards CAG as a precancerous condition for GC. Intestinal metaplasia and dysplasia are precancerous lesions of GC. The key to reducing GC mortality is early detection because this provides an opportunity to surgically remove precancerous lesions.

Currently, there are several approaches to screen for GC, such as gastroscopy, computed tomography, and serum tumor markers. Gastroscopy combined with pathological diagnosis is the gold standard for GC diagnosis, but it is invasive and relies largely on examiner expertise. In addition, early cancer screening equipment, such as magnifying endoscopy and the electronic endoscopy pigment method, are still not available in many medical institutions. Although imaging examination can provide helpful clinical information, it has limited value in the diagnosis of early GC (EGC) and cannot be used routinely as a monitoring examination because of the risks related to long-term radiation exposure. Meanwhile, it has been reported that clinical serum tumor markers, such as carcinoembryonic antigen (CEA), carbohydrate antigen 19-9 (CA19-9), and carbohydrate antigen 72-4 (CA72-4), have a sensitivity of 20% for EGC [3,4]. Hence, it is important to adopt a simple method with high sensitivity to compensate for the limitations of the abovementioned methods. Liquid biopsy is one such useful method.

One of the underlying foundations of liquid biopsy is the release of cell-free DNA (cfDNA) from apoptotic and necrotic cancer cells into the blood [5]. The isolated cfDNA contains the same molecular aberrations as solid tumors, such as mutations and methylation [6]. DNA methylation is usually an early event in carcinogenesis, and it has been observed in precancerous lesions [7]. From the perspective of etiology, *Helicobacter pylori* and Epstein–Barr virus infection may result in chronic inflammation of the gastric mucosa, which can induce GC by promoting promoter methylation [8]. A certain methylation status in a particular gene for noninvasive monitoring EGC development in affected individuals is invaluable, which is an unmet need nowadays.

Septin9 is a GTP/GDP-binding protein that is mainly responsible for cell division and chromosome segregation [9]. The methylation of SEPT9 (Septin9 gene) inhibits its normal expression, eventually disrupting cell division and leading to cancer. In colorectal cancer, methylated SEPT9 (mSEPT9) can be used as a tumor biomarker to diagnose the disease and predict the risk of prognosis, recurrence, and metastasis, and its value has been confirmed in clinical practice [10]. Since colorectal cancer and GC are both gastrointestinal tumors, they share some common molecular characteristics, and the value of mSEPT9 detection in the screening of GC has gradually attracted attention [11]. In 2013, a study first reported that the mSEPT9 level increased in the plasma of patients with both GC and colorectal cancer [12], suggesting that it may be useful in the diagnosis of GC. CSG, CAG, GU, and GP are common in outpatients and inpatients coming to hospitals. Considering the clinical implication and the sample accessibility, we selected the above subjects as comparison groups and conducted this study under the background of opportunistic screening, which is different from screening an average-risk population; the probability of GC detection by opportunistic screening is higher. We measured the plasma mSEPT9 level and compared the sensitivity and specificity of mSEPT9 to tumor markers. We also analyzed the correlation between mSEPT9 cycle threshold (Ct) values and clinicopathological parameters. This study may provide valuable information for the screening, diagnosis, and targeted therapy of GC.

## 2. Materials and Methods

### 2.1. Patient Recruitment and Ethical Consideration

A total of 182 pretreatment plasma specimens, which included those of 60 patients with GC, 39 with CSG, 27 with CAG (13 with intestinal metaplasia and 1 with mild dysplasia), 30 with GU, and 26 with GP, were collected from the Tianjin Union Medical Center from August 2020 to August 2021. All patients met the following criteria: (1) complete case data; (2) definitive gastroscopy and pathological diagnosis; (3) no history of malignant tumor in other organs; (4) plasma samples were collected before surgery, chemotherapy, or radiotherapy; and (5) completed the whole process of sample collection. The patients’ clinical characteristics, including sex, age, tumor location, tumor size, cTNM stage, differentiation, and Lauren type, were recorded. The cTNM staging of GC was based on the 8th edition of the American Joint Committee on Cancer. This study was approved by the moral and ethics committee of Tianjin Union Medical Center (YJ006/2020), and informed consent was obtained from all participants. 

### 2.2. Sample Collection, Processing, and Storage

A peripheral blood sample (10 mL) was collected using 10 mL BD Vacutainer^®^K2E (EDTA) anticoagulant tubes (BD, Devon, UK) for SEPT9 gene methylation assays. Blood samples were centrifuged for 12 min at 1350 ± 150 rcf, and the plasma was collected in a 15 mL centrifuge tube. The plasma was centrifuged again for 12 min at 1350 ± 150 rcf, and 3.5 mL supernatant was collected in a centrifuge tube. Blood samples were collected and processed at 2–8 °C on the same day within 8 h. Plasma samples were stored at −15 °C to −25 °C before subsequent cfDNA extraction. The SEPT9 gene methylation assay was performed within 2 weeks after the samples were collected.

### 2.3. cfDNA Extraction and Bisulfite Conversion

Plasma cfDNA extraction and bisulfite conversion were performed using a nucleic acid extraction kit (BioChain Science and Technology, Inc., Beijing, China) according to the manufacturer’s instructions. Briefly, the extraction of DNA contained in patient plasma is based on the binding of cfDNA to magnetic particles, and the unmethylated cytosine was transformed to uracil, whereas methylated cytosine remained unchanged. Finally, bisulfite-modified DNA (BisDNA) was obtained after washing and elution.

### 2.4. SEPT9 Gene Methylation Assay

The diagnostic kit for SEPT9 gene methylation is a qualitative assay for real-time polymerase chain reaction (qPCR) detection of mSEPT9 in BisDNA from human plasma samples. The assay was performed according to the manufacturer’s instructions. All kits were provided by BioChain Science and Technology, Inc., Beijing, China. Briefly, each BisDNA sample (patient sample, positive control, or negative control) was tested in triplicate. The PCR program was set as follows: activation at 94 °C for 20 min; 45 cycles at 62 °C for 5 s, 55.5 °C for 35 s, and 93 °C for 30 s; and cooling at 40 °C for 5 s.

ACTB (β-actin) served as an internal control to assess the quantity of input DNA and the validity of PCR amplification. Results were considered valid when the ACTB Ct was ≤34.8, and the external negative and positive controls met the validity criteria specified by the manufacturer. mSEPT9 Ct < 45 was considered a positive result, whereas mSEPT9 Ct ≥ 45 or an undetermined Ct value was considered a negative result.

Using different algorithms helps to improve the positive rate of detection. A previous study showed that the performance of mSEPT9 in colorectal cancer is affected by different algorithms. In this study, both 1/3 and 2/3 algorithms were used to evaluate its performance in GC; 1/3 algorithm: the test result for a patient sample was “POSITIVE” if at least one of three PCR replicates (1/3 or 2/3 or 3/3) were SEPT9 Positive. The test result for a patient sample was “NEGATIVE”, if all of three PCR replicates (0/3) were SEPT9 Negative. Based on the 2/3 algorithm, the test result for a patient sample was “POSITIVE” if at least two of three PCR replicates (2/3 or 3/3) were SEPT9 Positive. The test result for a patient sample was “NEGATIVE” if at least two of three PCR replicates (0/3 or 1/3) were SEPT9 Negative.

### 2.5. Detection of Serum Tumor Markers

CEA, CA19-9, and CA72-4 were all detected by the Department of Clinical Laboratory of Tianjin Union Medical Center by electrochemiluminescence immunoassay. A Roche E-70 electrochemiluminescence immunoassay analyzer and Roche E-70 special kit were used. CEA ≥ 5.0 ng/mL, CA19-9 ≥ 37.0 U/mL, and CA72-4 ≥ 6.0 U/mL were defined as positive. 

### 2.6. Statistical Analysis

Statistical analyses were performed using SPSS Version 26 (SPSS, Chicago, IL, USA) and GraphPad Prism version 8.3 (GraphPad Software, San Diego, CA, USA). The mean Ct values of mSEPT9 in the GC and benign gastric diseases (BGD) groups were compared using independent sample t-tests. The difference in the positive detection rate between mSEPT9 and serum tumor markers was evaluated using the McNemer test and Kappa test. The clinicopathological characteristics of the GC group were analyzed using the χ2 test or Fisher’s exact test. A binomial distribution was assumed for the calculation of the 95% confidence interval (CI). The receiver operating characteristic (ROC) curve was plotted using the mean Ct values of mSEPT9 from the GC and BGD groups. Ct values were set to 45 (maximum PCR cycle number) for the undetermined samples. The area under the ROC curve (AUC) was calculated. Statistical significance was set at a *p*-value of <0.05.

## 3. Results

### 3.1. The Methylation Status of SEPT9 in Plasma DNA within Different Groups

**Figure 1:**
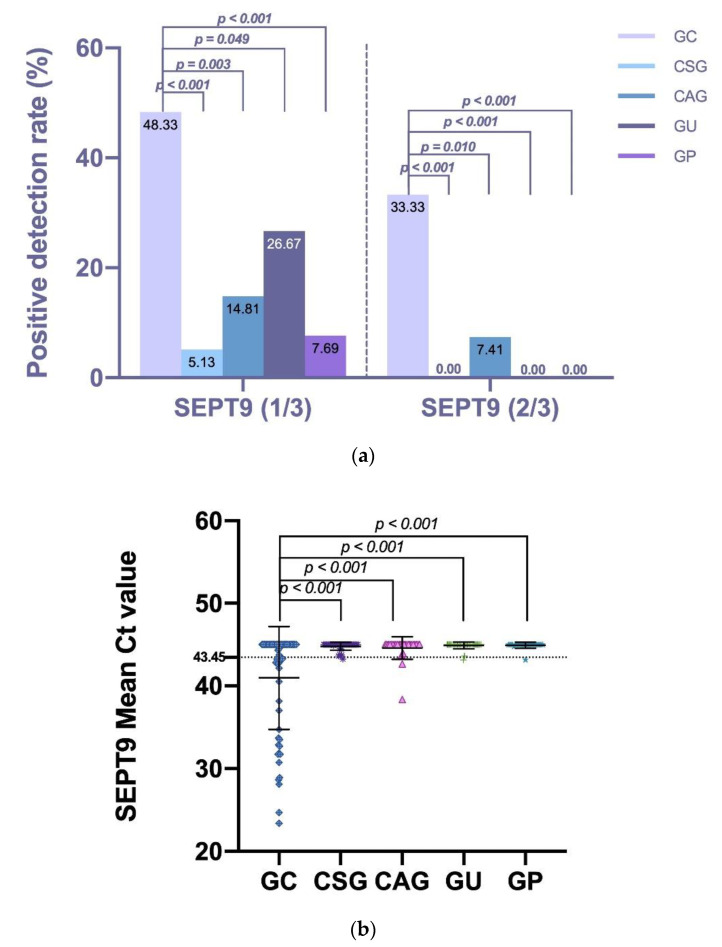
The positive rate and mean Ct value of mSEPT9 in each enrolled group. Different colors represent different groups. (**a**) The positive detection rate of mSEPT9 for each enrolled group based on the 2/3 or 1/3 algorithm. The positive detection rate was compared by the χ2 test or Fisher’s exact test. mSEPT9 level (1/3 and 2/3 algorithm) was significantly higher in the GC group than in the CSG, CAG, GU and GP groups. (**b**) The mSEPT9 mean Ct value for each enrolled group. The mean Ct value was compared by t tests. The mean mSEPT9 Ct value was significantly higher in the GC group than in the CSG, CAG, GU and GP groups. The mSEPT9 Ct cut-off value = 43.45 was calculated by the Youden index.

To determine whether the DNA methylation statue of SEPT9 in plasma samples had diagnostic value for GC, the positive rate of 60 patients with GC, 39 with CSG, 27 with CAG, 30 with GU, and 26 with GP was analyzed. Based on the 1/3 algorithm, mSEPT9 was identified in the pretreatment plasma of 29 patients with GC (48.33%), two patients with CSG (5.13%), four patients with CAG (14.81%), eight patients with GU (26.67%), and two patients with GP. The mSEPT9 level (1/3 algorithm) was significantly higher in the GC group than in the CSG (*p* < 0.001), CAG (*p* = 0.003), GU (*p* = 0.049), and GP (*p* < 0.001) groups. Based on the 2/3 algorithm, mSEPT9 was identified in the pretreatment plasma of 20 patients with GC (33.33%) and two patients with CAG (7.41%) but was not found in patients with CSG, GU or GP. Similarly, the mSEPT9 level (2/3 algorithm) was significantly higher in the GC group than in the CSG (*p* < 0.001), CAG (*p* = 0.010), GU (*p* < 0.001), and GP (*p* < 0.001) groups. Additional analysis of the mean mSEPT9 Ct values also showed significant results. The mean mSEPT9 Ct value for the GC, CSG, CAG, GU, and GP groups was 40.98 ± 6.21, 44.92 ± 0.36, 44.58 ± 1.35, 44.73 ± 0.53, and 44.92 ± 0.36 (all *p* values < 0.001), respectively (shown in Table 1 and Figure 1).

### 3.2. The Diagnostic Value of Plasma mSEPT9 in Patients with GC

To evaluate the diagnostic value of mSEPT9, the CSG, CAG, GU, and GP groups were treated as one BGD group. Based on the 1/3 algorithm, mSEPT9 had 48.33% sensitivity, 86.89% specificity, 64.44% positive predictive value (PPV), and 77.37% negative predictive value (NPV). Based on the 2/3 algorithm, mSEPT9 had 33.33% sensitivity, 98.36% specificity, 90.91% PPV, and 75.00% NPV (shown in Table 2).

**Figure 2:**
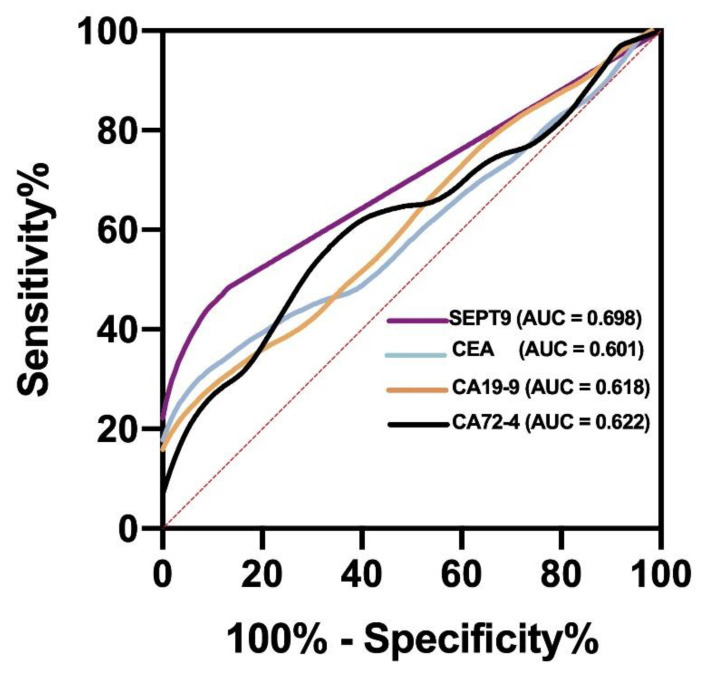
ROC curve of mSEPT9 and serum tumor markers. AUC (SEPT9) > AUC (CA72-4) > AUC (CA19-9) > AUC (CEA). AUC, the area under the ROC curve.

To achieve the observed test performance, ROC curve analysis was performed to evaluate the AUC, sensitivity, and specificity and to determine the best cut-off value of mSEPT9 for GC diagnosis. mSEPT9 had an AUC of 0.698 (95% CI, 0.609–0.787), sensitivity of 41.67%, and specificity of 95.90% at the Ct cut-off value of 43.45 (shown in Table 3 and Figure 2). These results showed that plasma mSEPT9 demonstrated a remarkable performance in the diagnosis of GC as compared to BGD. In addition, when the mSEPT9 Ct value is lower than 43.45, doctors should suggest gastroscopy to rule out malignant lesions. In this study, an mSEPT9 Ct value of <45 was considered positive, which was close to the cut-off value calculated using the Youden index.

### 3.3. A Comparison of the Predictive Power of mSEPT9, CEA, CA19-9, and CA72-4 for GC Detection

CEA was found in 17 of 60 patients with GC and 6 of 122 control patients. In addition, CA19-9 was found in 16 of 60 patients with GC and 8 of 122 control patients. Similarly, CA72-4 was found in 17 of patients with 60 GC and 14 of 122 control patients. Thus, the sensitivities of CEA, CA19-9, and CA72-4 were 28.33%, 26.67%, and 28.33%, respectively, which were all lower than those of mSEPT9 (both 1/3 and 2/3 algorithms) (shown in Table 2). Regarding AUC for GC, the area of mSEPT9 was larger than that of CEA, CA19-9, and CA72-4 (shown in Table 3 and Figure 2).

**Figure 3:**
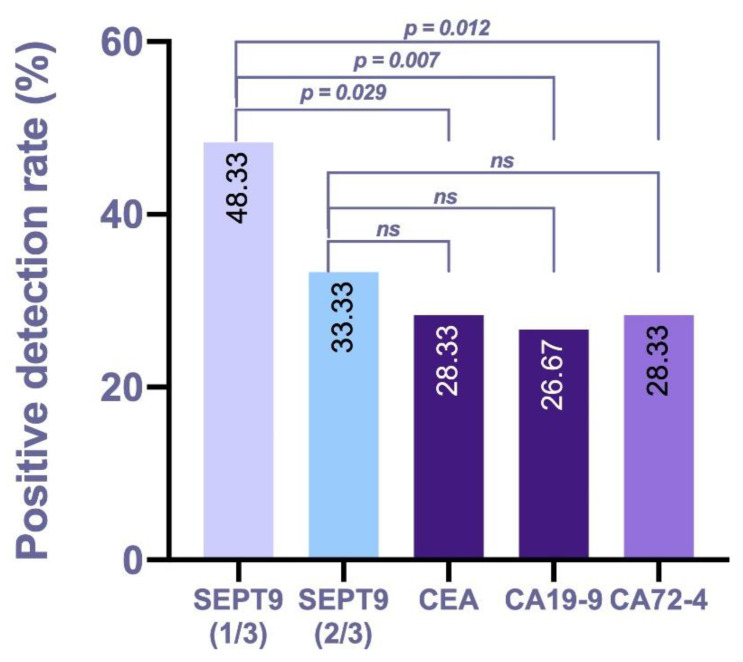
Positive rates of mSEPT9 and serum tumor markers in the GC group. The positive detection rate was compared by McNemer test. *ns*, not significant.

To compare the predictive power between mSEPT9 and serum tumor markers during the auxiliary diagnosis of GC, additional McNemer tests and kappa tests were conducted in the GC group, and the results showed that the overall agreement was poor between both tests (kappa ranged from 0.121 to 0.416), and the positive rate of mSEPT9 with the 1/3 algorithm was significantly higher than that of CEA (*p* = 0.029), CA19-9 (*p* = 0.007), and CA72-4 (*p* = 0.012). However, the positive rate of mSEPT9 with the 2/3 algorithm was not significantly higher than that of CEA, CA19-9, and CA72-4 (*p* > 0.05) (shown in Table 4 and Figure 3). These data indicate that mSEPT9 analyzed with the 1/3 algorithm was specific for GC detection and better than mSEPT9 with the 2/3 algorithm, CEA, CA19-9, and CA72-4.

### 3.4. Correlation of Pretreatment Plasma mSEPT9 with the Clinicopathological Characteristics of GC

**Figure 4:**
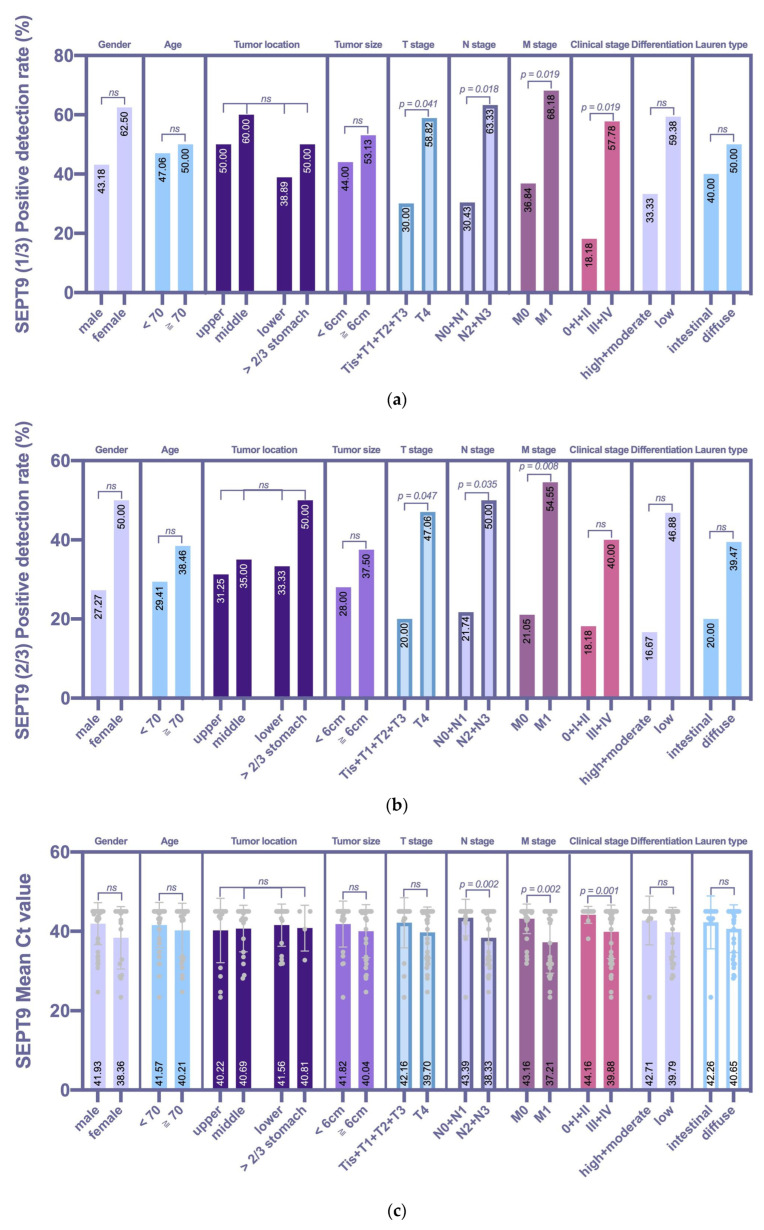
Correlations between mSEPT9 and the clinicopathologic characteristics of the GC group. (**a**) The 1/3 algorithm was compared by the χ2 test or Fisher’s exact test. GC patients with late T stage (*p* = 0.041), N stage (*p* = 0.018), M stage (*p* = 0.019), and clinical stage (*p* = 0.019) showed significantly higher mSEPT9 positivity than those in the early stages. (**b**) The 2/3 algorithm was compared by the χ2 test or Fisher’s exact test. GC patients with late T stage (*p* = 0.047), N stage (*p* = 0.035) and M stage (*p* = 0.008) showed significantly higher mSEPT9 positivity than those in the early stages. (**c**) The mean Ct value was compared by a t test or an F test. The mean Ct value was associated with N stage (*p* = 0.002), M stage (*p* = 0.002), and clinical stage (*p* = 0.001), but not with the T stage (*p* = 0.179).

Data on the clinicopathological characteristics of the GC group were collected to study the specific correlation between peripheral mSEPT9 and pathological manifestations (shown in Table 5 and Figure 4). Based on the 1/3 algorithm, the GC patients with late T stage (*p* = 0.041), N stage (*p* = 0.018), M stage (*p* = 0.019), and clinical stage (*p* = 0.019) showed significantly higher mSEPT9 positivity than those in the early stages. Based on the 2/3 algorithm, which was different from the 1/3 algorithm, mSEPT9 positivity was not associated with the clinical stage, although there was a trend that the positivity for stage 0/I/II was lower than that for stage III/IV. We further analyzed the mean Ct value of mSEPT9. The mean Ct value was also associated with N stage (*p* = 0.002), M stage (*p* = 0.002), and clinical stage (*p* = 0.001), but not with the T stage (*p* = 0.179). Although the analysis of the results of the above three methods was not exactly the same, they all indicated that mSEPT9 positivity was not associated with sex, age, tumor localization, tumor size, differentiation, and Lauren type. It seems that mSEPT9 pretreatment may be a significant tool for distinguishing between early and advanced GC.

## 4. Discussion

DNA methylation is the methylation of the 5′ carbon atom of cytosine in cytosine-guanine dinucleotide (CpG) to form 5-methylcytosine [13]. It is the most studied form of epigenetic modification. DNA methylation is usually an early event in carcinogenesis that has been detected in some precancerous lesions [7]. Abnormal DNA methylation occurs more frequently than genetic mutations, which can also be detected in circulating tumor DNA. This may, therefore, be an ideal and useful biomarker for the early detection and staging of cancer. In this study, we conducted opportunistic screening for patients with GC and BGD, such as CAG, CSG, GU, and GP. Our study shows that plasma mSEPT9 testing adds diagnostic and progression information and constitutes an effective ancillary method for the differential diagnosis of malignant, paramalignant, and benign gastric diseases.

SEPT9 plays a role in multiple cancers as either an oncogene or a tumor suppressor gene [14,15]. In colorectal cancer, classical Hodgkin lymphoma, nasopharyngeal carcinoma, breast tumors, head and neck squamous cell carcinoma, and glioblastoma, SEPT9 served as a tumor suppressor gene [14,16,17,18,19,20,21]. In clinical practice, mSEPT9 has been widely used in the diagnosis of colorectal cancer, and a study has reported that its level increased in the plasma of patients with both GC and colorectal cancer [12], suggesting that it may be useful in the diagnosis of GC. Previous studies focused on the expression of mSEPT9 in GC tissues. In Lee’s study, mSEPT9 was detected in 14 cancer tissues (56.0%) and four normal tissues (16.0%; *p* = 0.002) [22]. A study in China showed that 72.0% (59/82) of the gastric cancer tissue samples had significantly higher mSEPT9 than the corresponding adjacent normal tissue (22.2%, 2/9, *p* < 0.01) [23]. Considering the accessibility of sample acquisition, detecting DNA methylation in peripheral blood undoubtedly provides a new method for the noninvasive detection of GC.

However, previous observations on plasma samples have shown inconsistent results; the sensitivity of mSEPT9 in GC varies from 17% to 70%. In 2013, Lee et al. studied the plasma samples of 153 patients with GC and observed a sensitivity of 17.7% with a specificity of 90.6% [12]. In 2018, Fu et al., studied the diagnostic performance of mSEPT9 for colorectal cancer and found that mSEPT9 was positive in 70% (7/10) of patients with GC [24]. In 2020, Cao et al. demonstrated that the positivity of mSEPT9 in the EGC group was significantly higher than that of the BGD and control groups (28.4% vs. 6.1% vs. 5.3%), and mSEPT9 had an AUC of 0.616 [25]. In 2020, Song et al. found that mSEPT9 assay had a sensitivity of 47.7% and specificity of 92.3% with an AUC of 0.76 [22]. Similar to Song’s study, our results revealed a sensitivity of 48.33% for mSEPT9 with the 1/3 algorithm and 33.33% with the 2/3 algorithm (AUC = 0.698). The performance of mSEPT9 in colorectal cancer is affected by different algorithms [26,27]. Similarly, our study shows that the 1/3 algorithm exhibited better performance in GC detection than the 2/3 algorithm, but the false positive rate of the 1/3 algorithm in the BGD group was also higher than that in the 2/3 algorithm. Thus the 1/3 algorithm is recommended for screening purposes. When the mSEPT9 test with the 1/3 algorithm is positive, patients are recommended to have a gastroscopy examination. To the best of our knowledge, our study is the first to validate the role of different mSEPT9 algorithms in patients with GC and to show that mSEPT9 analyzed with the 1/3 algorithm exhibits better performance in disease detection than that with the 2/3 algorithm, which is recommended for screening purposes. Further, our study was conducted under the background of opportunistic screening with comprehensive comparison groups of GC.

However, the positive rate of GC was much lower than that of colorectal cancer. The reasons may include the following two points. First, GC exhibits growth patterns that differ from those of colorectal cancer, as the detection of mSEPT9 in blood is heavily dependent on the release of DNA from cancer cells into the blood. If the growth of cancer does not involve abundant angiogenesis and vessel invasion, the detection rate for mSEPT9 may be low [22]. Second, owing to the secretion of hydrochloric acid from parietal cells in gastric mucosa, stomach juice is strongly acidic, with a pH less than 3. A previous study showed that DNA is denatured by stomach acidity, which may help explain the low sensitivity of mSEPT9 in GC [28,29]. Future studies will investigate these possibilities.

In Lee’s study, plasma mSEPT9 was associated with TNM stage, distant metastasis, Lauren classification, and tumor border [12]. In Cao’s study, there was no correlation between the mSEPT9 positive rate and clinicopathological characteristics [25]. In Song’s study, mSEPT9 was only associated with cancer location [22]. Inconsistent with these studies, we analyzed the mean mSEPT9 Ct value and mSEPT9 positive rate with different algorithms and found that mSEPT9 was associated with the T stage, N stage, M stage, and clinical stage, but not with sex, age, tumor localization, tumor size, differentiation, and Lauren type. As the depth of invasion (T), lymph node metastasis (N), and distant metastasis (M) were all positively correlated with the stage of GC, our results suggest that mSEPT9 is a marker of cancer progression.

The different results may be explained from a few aspects. First, different kits, algorithms, and the composition of GC in each study affected the test performance. In the early stage of GC, the invasive behavior is not yet observed in most of the patients. As the disease advances, tumor cells gradually infiltrate deep tissues, blood vessels, and lymph nodes, and more mSEPT9 will be released into circulation. Second, the small sample size affected the experimental results. One experiment included only 10 GC patients, with a sensitivity of 70% (7/10) [24]. Third, it is noteworthy that the SEPT9 gene has a complex genomic architecture with 18 distinct transcripts generated by alternative splicing [30], and SEPT9 gene transcription expression patterns differ in tumors. For example, evidence has shown that the hypermethylation of the v2 transcript of the SEPT9 gene occurs in almost 100% of colorectal cancer tissues [14], elevated levels of SEPT9 v1 and v4 are common in ovarian cancer [31], and SEPT9 v1 is highly expressed and v2 is less expressed in breast cancer [32]. Considering tissue specificity and alternative splicing, the gene methylation corresponding to a certain transcript must be the point of focus, but current kits cannot meet the requirement; therefore, the results varied greatly and were not as good as expected. Future research needs to explore the specific splicing of GC and improve the corresponding kit.

Notably, Cao et al. assessed mSEPT9 for the noninvasive diagnosis of EGC and found that the positive rate of mSEPT9 was higher than that of CA72-4, CA12-5, CEA, and CA19-9 [25], which is consistent with our study. The positive rate of mSEPT9 was superior to that of CEA, CA19-9, and CA72-4. In Cao’s study, in which mRNF180 was combined with mSEPT9, and in Song’s study, in which mSEPT9 was combined with CA72-4, the sensitivity of the combined test improved, and the specificity remained satisfactory [22,25]. One strategy for improving GC diagnosis is to combine multiple methylation biomarkers, such as P16, CDH1, MGMT, RARB, and RNF180 [33,34]. Further studies are warranted to identify more biomarkers and refine the best panel for GC detection. Improving the detection accuracy by optimizing the methylation detection technology and exploring the specific signal pathway mechanisms underlying methylation oncogenic effects will be the focus of future investigations.

## 5. Conclusions

In summary, the performance of plasma mSEPT9 was better than that of CEA, CA19-9, and CA72-4 and could be considered a useful and noninvasive biomarker for diagnosing GC and distinguishing between benign and malignant diseases of the stomach, especially for patients with poor basic conditions who are unable to tolerate gastroscopy. In addition, mSEPT9 was associated with T stage, N stage, M stage, and clinical stage, and this can be used to evaluate the progression of GC.

## Figures and Tables

**Table 1:** The mean Ct value and positive rate of mSEPT9 in each enrolled group.

Groups	*n*	Positive (1/3)		Positive (2/3)		Mean Ct Value	
		Number	%	Number	%	Mean	Std. Deviation
GC	60	29	47.54	20	33.33	40.98	6.21
CSG	39	2	5.13	0	0	44.92	0.36
CAG	27	4	14.81	2	7.41	44.58	1.35
GU	30	8	26.67	0	0	44.73	0.53
GP	26	2	7.69	0	0	44.92	0.36
*p* *		<0.001		<0.001		<0.001	
*p* **		0.003		0.010		<0.001	
*p* ***		0.049		<0.001		<0.001	
*p* ****		<0.001		<0.001		<0.001	

p * compared with CSG; *p* ** compared with CAG; p *** compared with GU; p **** compared with GP. GC, gastric cancer; CSG, chronic superficial gastritis; CAG, chronic atrophic gastritis; GU, gastric ulcer; GP, gastric polys.

**Table 2:** Sensitivity, Specificity, PPV, and NPV of mSEPT9 and serum tumor markers.

	Sensitivity (95% CI)	Specificity (95% CI)	PPV (95% CI)	NPV (95% CI)
SEPT9 (1/3)	48.33 (35.40–61.48)	86.89 (79.28–92.09)	64.44 (48.73–77.71)	77.37 (69.28–83.89)
SEPT9 (2/3)	33.33 (22.02–46.79)	98.36 (93.61–99.72)	90.91 (69.38–98.41)	75.00 (67.43–81.35)
CEA	28.33 (17.82–41.64)	95.08 (89.15–97.99)	73.91 (51.31–88.92)	72.96 (65.24–79.54)
CA19-9	26.67 (16.45–39.89)	93.44 (87.08–96.92)	66.67 (44.69–83.57)	72.15 (64.37–78.84)
CA72-4	28.33 (17.82–41.64)	88.52 (81.18–93.35)	54.84 (36.30–72.22)	71.52 (63.51–78.42)

PPV, positive predictive value; NPV, negative predictive value; CEA, carcinoembryonic antigen; CA19-9, carbohydrate antigen 19-9; CA72-4, carbohydrate antigen 72-4.

**Table 3:** Area under the receiver operating characteristic curve (AUC).

Groups	AUC (95% CI)	Std. Error	Asymptotic Sig.	Cut-off Value	Sensitivity (95% CI)	Specificity (95% CI)
SEPT9	0.698 (0.609–0.787)	0.045	<0.0001	43.45	41.67 (30.06–54.27)	95.90 (90.76–98.24)
CEA	0.601 (0.507–0.695)	0.048	0.0268	4.54	31.67 (21.31–44.23)	94.26 (88.63–97.19)
CA19-9	0.618 (0.529–0.708)	0.046	0.0097	22.96	35.00 (24.17–47.64)	86.07 (78.81–91.11)
CA72-4	0.622 (0.531–0.713)	0.046	0.0076	2.12	60.00 (47.37–71.43)	66.39 (57.62–74.16)

**Table 4:** A comparison of the positive rates of mSEPT9 and serum tumor markers in the GC group.

	Positive Rate	p *	p **
SEPT9 (1/3)	48.33% (29/60)	-	-
SEPT9 (2/3)	33.33% (20/60)		-
CEA	28.33% (17/60)	0.029 (kappa value = 0.121)	0.664 (kappa value = 0.182)
CA19-9	26.67% (16/60)	0.007 (kappa value = 0.289)	0.454 (kappa value = 0.368)
CA72-4	28.33% (17/60)	0.012 (kappa value = 0.324)	0.607 (kappa value = 0.416)

p * compared with SEPT9 (1/3); p ** compared with SEPT9 (2/3).

**Table 5:** Correlations between mSEPT9 and the clinicopathologic characteristics of the GC group.

Clinicopathological Characteristics	SEPT9								
	*n*	Positive (1/3)	Negative (1/3)	*p* (1/3)	Positive (2/3)	Negative (2/3)	*p* (2/3)	Mean Ct Value	*p*
**Sex**				0.185			0.099		0.107
Male	44	19	25		12	32		41.93	
Female	16	10	6		8	8		38.36	
**Age (years)**				0.821			0.461		0.408
<70	34	16	18		10	24		41.57	
≥70	26	13	13		10	16		40.21	
**Tumor location**				0.639			0.906		
Upper third	16	8	8		5	11		NA	
Middle third	20	12	8		7	13		NA	
Lower third	18	7	11		6	12		NA	
>2/3 stomach	4	2	2		2	2		NA	
NA	2	0	2		0	2			
**Tumor size (cm)**				0.494			0.450		0.296
<6.0	25	11	14		7	18		41.82	
≥6.0	32	17	15		12	20		40.04	
NA	3	1	2		1	2			
**T stage**				0.041			0.047		0.179
Tis + T1 + T2 + T3	20	6	14		4	16		42.16	
T4	34	20	14		16	18		39.70	
NA	6	3	3		0	6			
**N stage**				0.018			0.035		0.002
N0 + N1	23	7	16		5	18		43.39	
N2 + N3	30	19	11		15	15		38.33	
NA	7	3	4		0	7			
**M stage**				0.019			0.008		0.002
M0	38	14	24		8	30		43.16	
M1	22	15	7		12	10		37.21	
NA	0	0	0		0	0			
**Clinical stage**				0.019			0.294		0.001
0 + I + II	11	2	9		2	9		44.16	
III + IV	45	26	19		18	27		39.88	
NA	4	1	3		0	4			
**Differentiation**				0.124			0.090		0.171
High + moderate	12	4	8		2	10		42.71	
low	32	19	13		15	17		39.79	
NA	16	6	10		3	13			
**Lauren type**				0.727			0.459		0.466
Intestinal	10	4	6		2	8		42.26	
Diffuse	38	19	19		15	23		40.65	
NA	12	6	6		3	9			

## Data Availability

The data used during the present study are available from the corresponding author upon reasonable request.

## Abbreviations

GC	gastric cancer
CSG	chronic superficial gastritis
CAG	chronic atrophic gastritis
GU	gastric ulcer
GP	gastric polys
CI	confidence interval
CEA	carcinoembryonic antigen
CA19-9	carbohydrate antigen 19-9
CA72-4	carbohydrate antigen 72-4
EGC	early gastric cancer
cfDNA	cell-free DNA
SEPT9	Septin9 gene
mSEPT9	methylated SEPT9
Ct	cycle threshold
BisDNA	bisulfite-modified DNA
qPCR	qualitative assay for real-time polymerase chain reaction
BGD	benign gastric diseases
ROC	receiver operating characteristic curve
AUC	the area under the ROC curve
PPV	positive predictive value
NPV	negative predictive value
CpG	cytosine–guanine dinucleotide
